# Hepatocyte Growth Factor Modification Enhances the Anti-Arrhythmic Properties of Human Bone Marrow-Derived Mesenchymal Stem Cells

**DOI:** 10.1371/journal.pone.0111246

**Published:** 2014-10-31

**Authors:** Jian Zhang, Lin-Lin Wang, Wei Du, Yi-Chao Yu, Wei-Zhu Ju, Yi-Long Man, Xiao-Rong Li, Yan Chen, Zi-Dun Wang, Wei-Juan Gu, Feng-Xiang Zhang, Hua Wang, Chu-Tse Wu, Ke-Jiang Cao

**Affiliations:** 1 Department of Cardiology, the First Affiliated Hospital of Nanjing Medical University, Nanjing, People's Republic of China; 2 Department of Cardiology, the Second Affiliated Hospital of Nanjing Medical University, Nanjing, People's Republic of China; 3 Department of Experimental Hematology, Beijing Institute of Radiation Medicine, Beijing, People's Republic of China; University of Cincinnati, College of Medicine, United States of America

## Abstract

**Background/Aims:**

Chronic myocardial infarction (MI) results in the formation of arrhythmogenic substrates, causing lethal ventricular arrhythmia (VA). We aimed to determine whether mesenchymal stem cells (MSCs) carrying a hepatocyte growth factor (HGF) gene modification (HGF-MSCs) decrease the levels of arrhythmogenic substrates and reduce the susceptibility to developing VA compared with unmodified MSCs and PBS in a swine infarction model.

**Methods:**

The left descending anterior artery was balloon-occluded to establish an MI model. Four weeks later, the randomly grouped pigs were administered MSCs, PBS or HGF-MSCs via thoracotomy. After an additional four weeks, dynamic electrocardiography was performed to assess heart rate variability, and programmed electrical stimulation was conducted to evaluate the risk for VA. Then, the pigs were euthanized for morphometric, immunofluorescence and western blot analyses. Results: The HGF-MSC group displayed the highest vessel density and Cx43 expression levels, and the lowest levels of apoptosis, and tyrosine hydroxylase (TH) and growth associated protein 43 (GAP43) expression. Moreover, the HGF-MSC group exhibited a decrease in the number of sympathetic nerve fibers, substantial decreases in the low frequency and the low-/high- frequency ratio and increases in the root mean square of successive differences (rMSSD) and the percentage of successive normal sinus R-R intervals longer than 50 ms (pNN50), compared with the other two groups. Finally, the HGF-MSC group displayed the lowest susceptibility to developing VA.

**Conclusion:**

HGF-MSCs displayed potent antiarrhythmic effects, reducing the risk for VA.

## Introduction

Ventricular arrhythmia (VA), particularly including ventricular tachycardia (VT) and ventricular fibrillation (VF), is a challenging complication during myocardial infarction (MI). A large amount of data suggest that sustained VT and VF are responsible for the majority of sudden cardiac death (SCD), which is predominantly caused by coronary heart disease [1]. Therefore, candidate interventions that may reduce the risk for VA represent encouraging study topics.

Despite the limitations of current clinical antiarrhythmic interventions, such as the limited effect of antiarrhythmic drugs and the unaffordable cost of implantable cardioverter defibrillator (ICD), a variety of stem cell treatments have been developed. It has been reported that MSCs not only exert a cardioprotective effect but may also decrease the high risk of VA following MI [2], [3]. This cell population has been widely used for cellular replacement therapy and tissue engineering because of the ease of its collection and transfection with exogenous genes [4]. However, most MSCs have been found to exhibit poor viability several days after cell engraftment, limiting their reparative effects [5].

It has been discovered that MSCs strongly express c-met, the well-characterized receptor of hepatocyte growth factor (HGF). HGF, originally recognized as a pro-angiogenic factor and an endothelial chemoattractant [6], [7], has been applied in cardiac ischemic diseases, as it induces neovascularization in ischemic tissues [8]. Previous results have also demonstrated that HGF protects MSCs from apoptosis [9], facilitating cell survival and proliferation after MSC transplantation [10]. This evidence renders HGF gene modification an ideal strategy for MSC treatment [11].

“Arrhythmogenic substrate” has been proposed in recent years in the field of electrophysiological study, especially for clinical arrhythmias. Arrhythmogenic substrate refers to the basis mediating the cardiac electrical activity that are likely to facilitate arrhythmias. The potential arrhythmogenic substrates includes the impaired myocardium, the electrical coupling disturbance, the conduction abnormality, the autonomic unbalance, the ischemic and fibrotic tissue, the body fluid and the endocrine factors. In this study, we postulated that HGF modification might endow transplanted MSCs with a more potent capacity to repair pro-arrhythmic substrates and a stronger potential to decrease the susceptibility to developing VA in a swine MI model.

## Materials and Methods

### Ethics Statement

Healthy male swine averaging 3 months of age (weighing 30±5 kg) were supplied by the Jiangsu Provincial Academy of Agricultural Sciences. All procedures were performed in accordance with institutional guidelines and were approved by the Institutional Animal Care and Use Committee of Nanjing Medical University.

### Cell Culture and Preparations

Prepared MSCs carrying an HGF gene modification (HGF-MSCs) were kindly provided by Professor Wu and his group (Beijing Institute of Radiation Medicine, Chinese Academy of Military Medical Sciences, Beijing, China), who also advised us regarding their use. Human MSCs were isolated, expanded in culture and transduced with an adenovirus carrying human HGF (Ad-HGF) as previously described [10]–[14], according to the ethical standards of their local ethics committee.

### Establishment of a Chronic Ischemia Model

**Figure 1:**
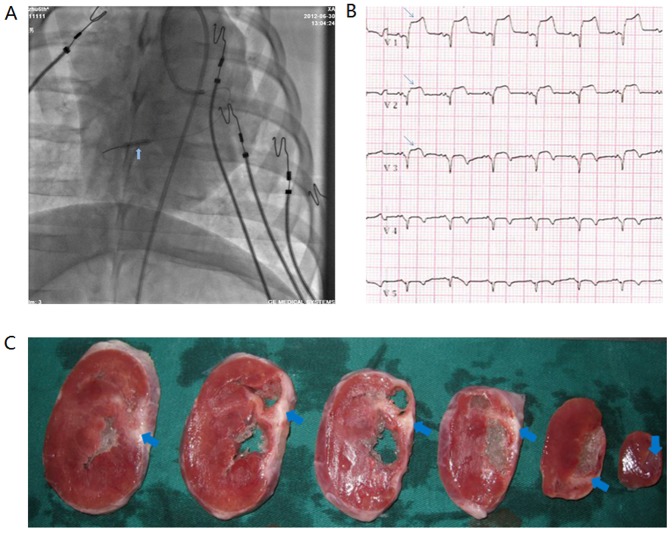
Model establishment and verification. (A) Balloon occlusion was performed to induce MI. (B) A substantial elevation of the ST segment was detected based on ECG. (C) TTC-positive staining demonstrated successful model establishment.

A total of 36 pigs were initially sedated using ketamine (10 mg/kg) and then maintained under anesthesia using a 3% sodium pentobarbital solution. Next, under fluoroscopy, a balloon-tipped coronary angioplasty catheter was percutaneously advanced over a guide wire to the distal LAD. Then, the balloon was inflated to a completely occlusive pressure for 90 minutes to induce transmural cardiac infarction (Fig. 1A). Following the successful establishment of a swine model in which the heart was ischemically impaired, the swine (n = 28) were randomly allocated to three groups as follows: HGF-MSC group (n = 9): these animals received HGF-MSC transplantation four weeks after LAD occlusion; phosphate buffer solution (PBS) group (n = 10): these animals received PBS injection four weeks after LAD occlusion; MSC group (n = 9): these animals received MSC transplantation four weeks after LAD occlusion.

### Cell Labeling

To observe HGF-MSCs in the impaired porcine heart after engraftment, 5-ethynyl-2′-deoxyuridine (EdU) was applied as a marker to label the HGF-MSCs in vitro during culture preparation. Samples from the infarcted border zone (IBZ) of the HGF-MSC group were examined using an EdU kit (Ruibo, Guangzhou, China).

### HGF-MSC Transplantation In Vivo

Four weeks after the induction of ischemia, open-chest operations were performed. After intubation, general anesthesia was maintained using sodium pentobarbital, and analgesia was maintained via hourly intravenous administration of fentanyl. Succinylcholine, a muscle relaxant, was administered every twenty minutes during the open-chest surgery. The pigs in the HGF-MSC and MSC groups received a total intramyocardial injection of 5×10^7^ HGF-MSCs or MSCs, respectively, suspended in 2 ml of sterile PBS, which were scattered at 10 sites in the IBZ of the infarction. The same volume of pure PBS was injected at the corresponding sites in the PBS group.

### Two-Hour Dynamic Electrocardiography (ECG)

The methods are detailed in Text S1.

### Electrophysiological Examination

The methods are detailed in Text S1.

### Animal Euthanasia and Specimen Processing

Once the indicated live animal experiments had been performed, all of the pigs were euthanized using a lethal dose of pentobarbital sodium (100 mg/kg) via intravenous injection. Then, their hearts were extracted from the porcine thorax. The left ventricular myocardium within 20 mm of the scar margin was defined as the IBZ [15]. The myocardial region more than 20 mm from the scar margin was defined as the remote zone, and the region within the infarcted scar was recognized as the infarct zone. The isolated individual tissue samples were packaged in a freezing tube and preserved in liquid nitrogen at −196°C for further applications.

### Western Blot and Immunofluorescence Analyses

The expression of connexin 43 (Cx43), tyrosine hydroxylase (TH), growth associated protein 43 (GAP43), acetylcholinesterase (AChE), Bcl-2 and Bax was detected via western blotting of the total protein fraction prepared from the IBZ at eight weeks post-infarction. (Detailed in Text S1).

### TUNEL Assay

To label cell death in situ, the Terminal dUTP nick end-labeling (TUNEL) assay was performed using an In Situ Cell Death Detection Kit, POD (11684817910, Roche). The apoptotic index was calculated in five fields in each section as: (apoptotic nuclei/number of total nuclei) X 100.

### Triphenyltetrazolium chloride (TTC) Staining

TTC staining was used to determine the infarcted and non-infarcted portion. The protocol is detailed in Text S1.

### Statistical Analysis

The continuous variables were expressed as the mean ± standard deviation. Student's t-test was performed to compare two groups. The categorical variables were expressed as percentages, and the Chi-square test was applied to compare the groups. *P*<0.05 was considered to be statistically significant.

## Results

### Establishment of the Chronic MI Model

The confirmation of successful model establishment depended on substantial ST segment elevation based on ECG, at least in leads V1–V3 (Fig. 1B), resulting from the complete occlusion of the distal LAD for 90 minutes (Fig. 1A). Eight pigs experienced repeated refractory VT or VF during the coronary plugging phase and ultimately died. For further evidence of successful model establishment, all pigs underwent ventriculography at 4 weeks post-infarction, and ventricular aneurysm was detected. The confirmed authentication of successful model establishment was revealed by positive TTC staining of the isolated swine heart in vitro (Fig. 1C).

### Confirmation of the Viability of the HGF-MSCs

**Figure 2:**
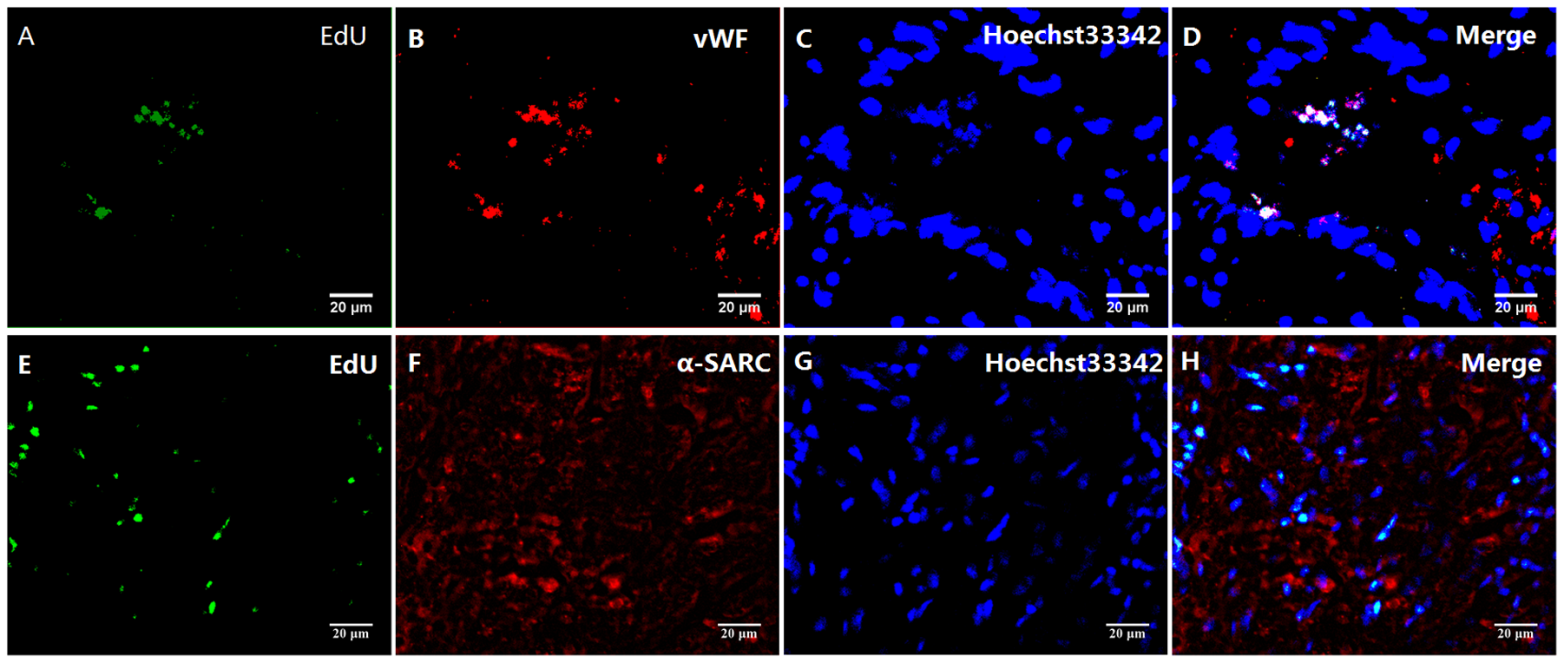
The fate of engrafted HGF-MSCs. (A–D) Double-immunofluorescence staining for viable HGF-MSCs in the infaction border zone four weeks after transplantation. The merged fluorescence indicates colocalization of EdU and vWF, suggesting that the HGF-MSCs exhibited a tendency to differentiate into vascular endothelial cells. (E–H) There was a lack of evidence of cardiomyogenic differentiation based on the absence of α-SARC/EdU colocalization.

The HGF-MSCs delivered to the damaged myocardium partially survived and displayed redistribution, proliferation and differentiation, which were detected based on green fluorescence following EdU kit treatment. White regions indicated double-staining of both EdU (green) and vWF (red) (Fig. 2 A–D), suggesting that the HGF-MSCs exhibited the potential to differentiate into vascular endothelial cells, although there was a lack of evidence supporting the cardiomyogenic differentiation of HGF-MSCs in this experiment (Fig. 2 E–H).

### Alteration of Vascularization and Apoptosis

**Figure 3:**
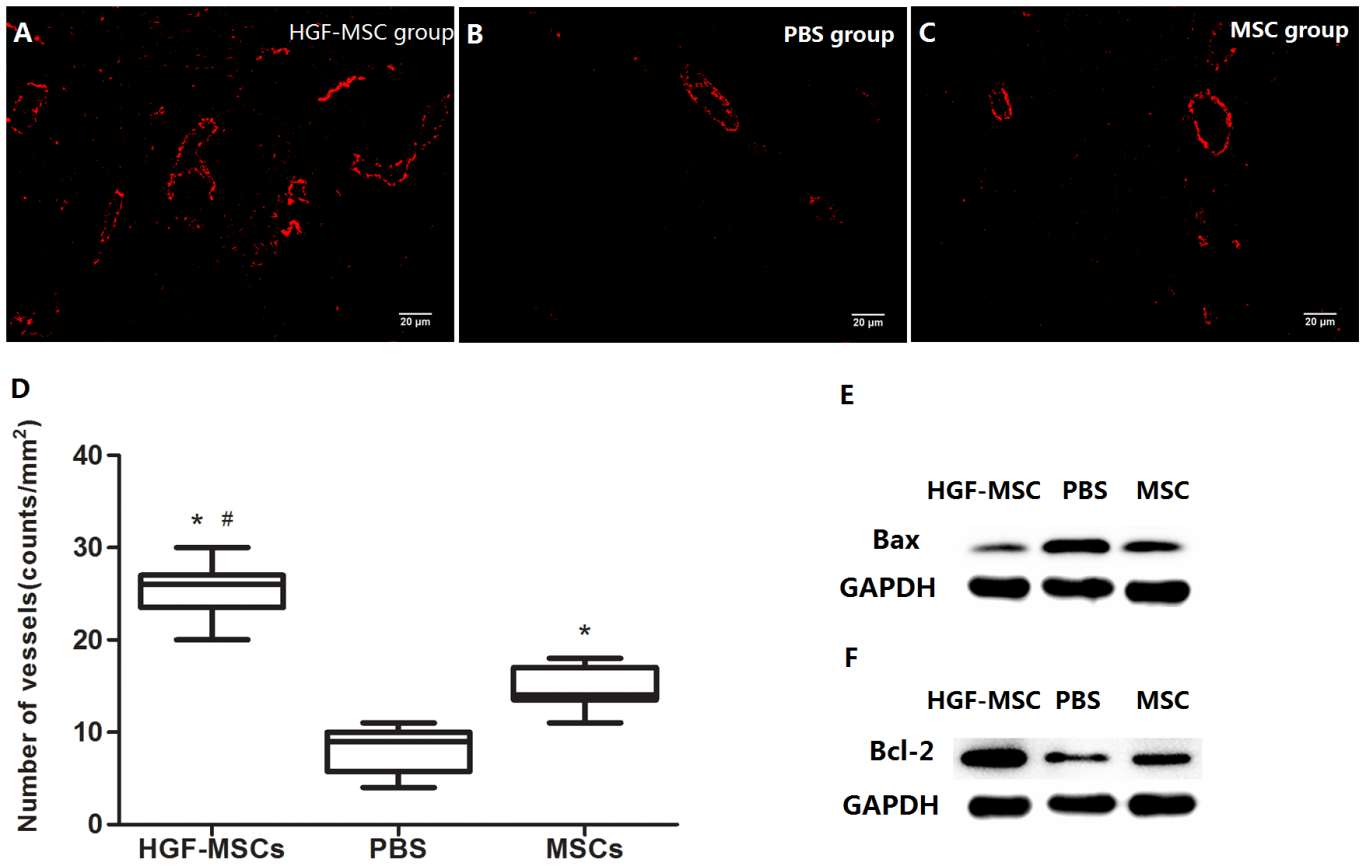
Alterations of vascularization and apoptosis. (A–C) Immunofluorescence staining to detect vascularization (vWF-positive staining) in the different experimental groups. (D) Quantification of the number of vWF-positive vessels at a 400X magnification in the different groups. *, *P*<0.01 vs. the PBS group; #, *P*<0.01 vs. the MSC group. (E–F) There was a significant decrease in the Bax protein expression level and a significant increase in the Bcl-2 expression level in the HGF-MSC group compared with the other two groups.

One potential beneficial effect of HGF-MSC transplantation was investigated by evaluating the difference in the vascularization of the post-MI hearts after treatment. Immunofluorescence staining for vWF indicated significant angiogenesis, and a higher vessel density was present in the IBZ of the HGF-MSC-treated hearts than the MSC- and PBS-treated hearts (Fig. 3 A–C), although the MSC group displayed a significant angiogenic effect compared with the PBS group. Quantitative analysis of the number of vWF-positive vessels per high-power field (40×) revealed a significantly greater vessel density in the HGF-MSC group than in the MSC and PBS groups (Fig. 3D). In addition, the HGF-MSC group displayed a larger decrease in Bax expression levels and increase in Bcl-2 expression levels than the MSC group when they were respectively compared with the PBS group (Fig. 3 E–F). The TUNEL assay results showed that only 1.29% nuclei were TUNEL-positive in the HGF-MSC group, whereas 2.00% nuclei stained TUNEL-positive in the MSC group (Fig. S1, *P*<0.05). When compared with the PBS group, the apoptotic index in both HGF-MSC and MSC groups displayed a remarkable decrease (Fig. S1, *P*<0.01). This proved that HGF overexpression could protect cardiomyocytes from apoptosis.

### Autonomic Nerve Spatial Re-innervation and Relevant Protein Expression Levels

**Figure 4:**
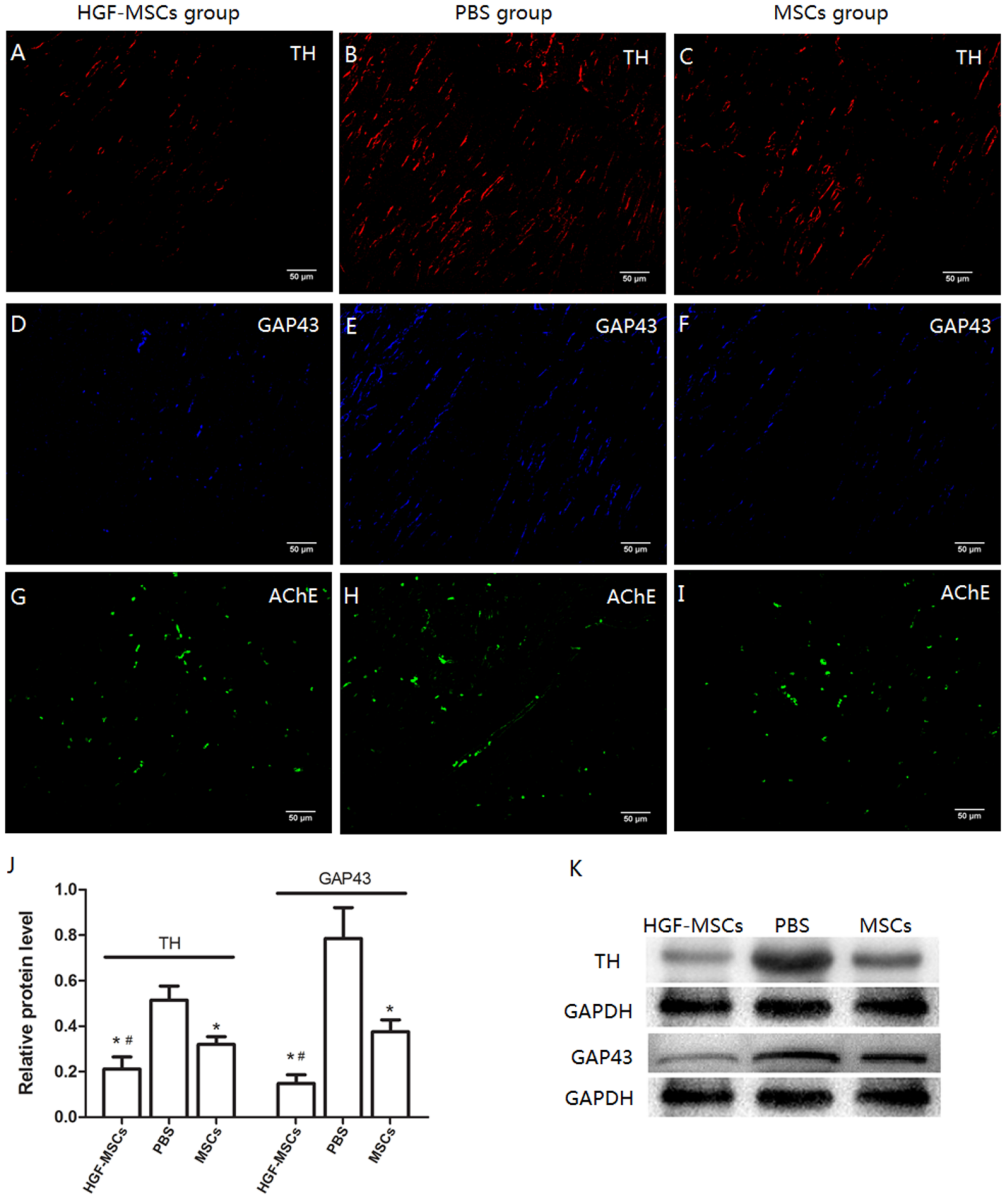
Neural remodeling. (A–C) Sympathetic nerve fibers were detected based on TH-positive immunostaining. (D–F) The GAP43 expression profile was specific for nerve sprouting. (G–I) The AChE-positive fibers indicated the parasympathetic distribution. MI-induced sympathetic sprouting and hyper-innervation were significantly attenuated in the HGF-MSC group compared with the PBS group. These effects appeared to be weaker between the MSC and PBS groups. However, the parasympathetic nerves displayed no substantial differences between the groups. (J–K) Western blot analysis confirmed a decrease in TH and GAP43 protein expression levels in the HGF-MSC group compared with the other two groups. A similar but smaller effect was detected in the MSC group compared with the PBS group. All values were normalized to that of the control. *, *P*<0.01 vs. the PBS group; #, *P*<0.01 vs. the MSC group.

GAP43, a protein expressed in the growth cones of sprouting axons, is a marker of nerve sprouting. TH is a marker of sympathetic nerves. The detected TH-immunostained nerve fibers appeared to be oriented along the longitudinal axis of adjacent myofibers. The TH-positive density was significantly reduced in the HGF-MSC and MSC groups compared with the PBS group (Fig. 4 A–C and Table 1). However, the pigs in the HGF-MSC-treated group displayed a lower TH-positive density in the IBZ than those in the MSC-treated group. Similar to the results obtained for TH, GAP43 immunostaining was significantly reduced in the HGF-MSC and MSC groups compared with the PBS group. Overall, the GAP43-positive nerve density was even more attenuated in the HGF-MSC group than in the MSC group (Fig. 4 D–F and Table 1). However, no significant difference in the AChE-immunostaining signal was detected (Fig. 4 G–I and Table 1). The differences in protein expression between the groups were further determined via western blot analysis (Fig. 4 J–K).

**Table 1:** Density of Nerve Staining in the Infarction Border Zone at 4 Weeks after Cell Delivery.

Marker	HGF-MSC group	PBS group	MSC group
TH (µm^2^/mm^2^)	_2863.11±122.48_^ab^	_6532.50±371.26_	_3324.44±150.08_ a
GAP43 (µm^2^/mm^2^)	_1094.78±133.23_^ab^	_6419.40±647.53_	_1777.00±453.13_ a
AChE (µm^2^/mm^2^)	_2497.89±359.86_	_2222.00±215.78_	_2460.00±239.14_

a , *P*<0.01 vs. the PBS group;

b , *P*<0.01 vs. the MSC group.

### Electrical Remodeling

**Figure 5:**
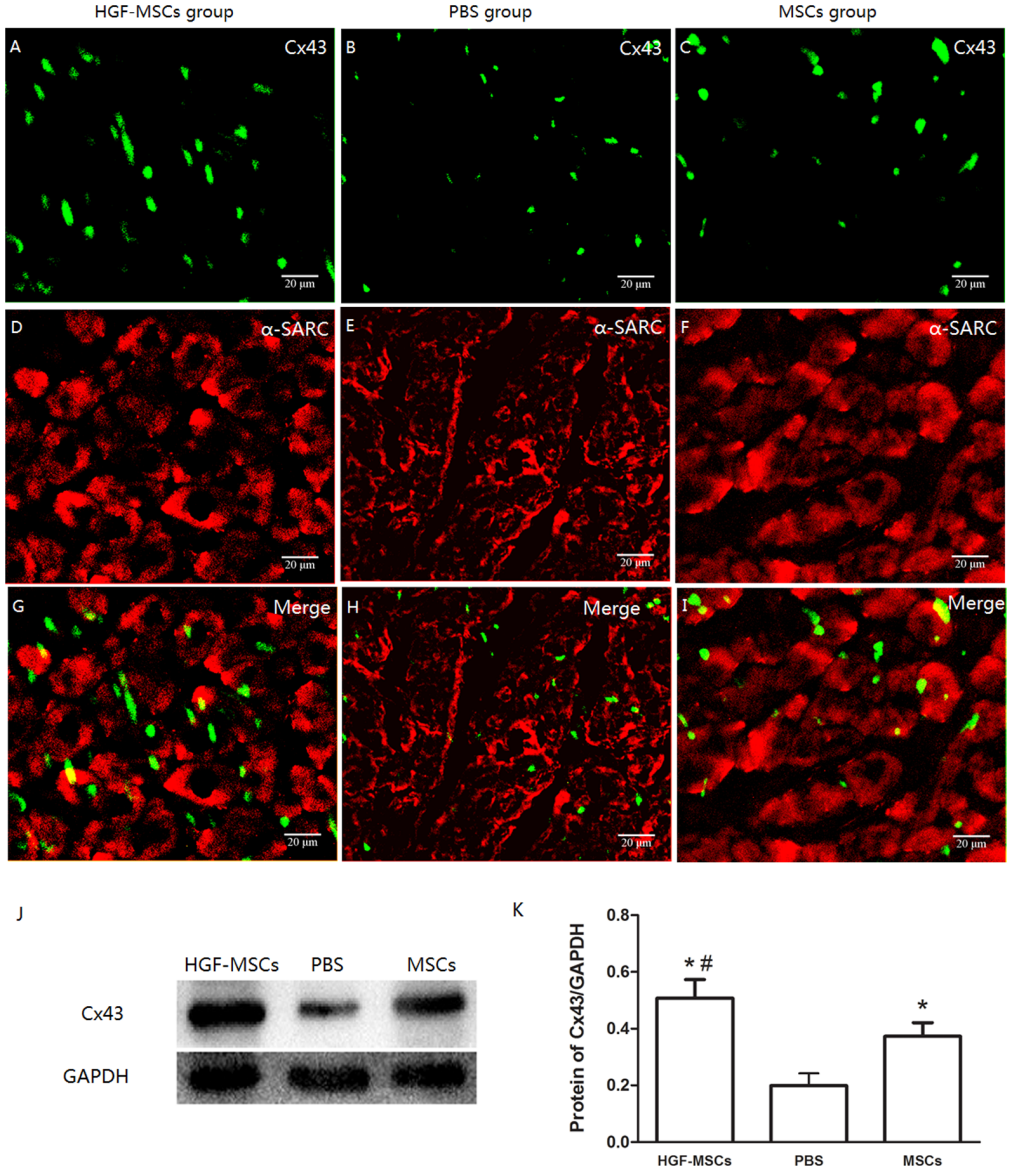
Electrical remodeling. (A–I) The myocardial infarction-induced disturbance of Cx43 expression, including lateralization and a reduction in area, was significantly restored and ameliorated, respectively, by HGF-MSC transplantation. MSC engraftment resulted in a similar but significantly weaker effect. (J–K) Cx43 expression levels were compared between the groups via western blot analysis. *, *P*<0.01 vs. the PBS group; #, *P*<0.01 vs. the MSC group.

Cx43 is the major gap junction-associated protein. In the IBZ of post-MI hearts, Cx43 expression is typically disturbed, including showing lateralization and a reduction of the area of expression. Here, although these aspects of Cx43 expression in the IBZ of the HGF-MSC group were markedly ameliorated and normalized, respectively, in the myocardium compared with the PBS group (Fig. 5), these effects were significantly reduced and not detected, respectively, in the MSC group (Fig. 5).

### Dynamic ECG Monitoring

Heart rate variability (HRV) has gained wide-spread acceptance as a clinical tool for evaluating cardiac autonomic changes in patients [16], [17]. In the present study, all of the examined HRV indices were evaluated via dynamic ECG. As summarized in Table 2, four weeks after cell transplantation, no significant difference in the standard deviation of the normal-to-normal R-R intervals (SDNN), the coefficient of variance (CV), the standard deviation of the averaged normal-to-normal R-R intervals (SDANN) or high frequency (HF) was observed between the groups (*P*>0.05). The HGF-MSC group exhibited a substantial enhancement of the root mean square of successive differences (rMSSD) and the percentage of successive normal sinus R-R intervals longer than 50 ms (pNN50), along with a considerable decrease in low frequency (LF) and the LF/HF ratio, compared with the MSC and PBS groups. However, these effects were significantly decreased in the MSC and PBS groups, indicating that the modulatory activity of the ANS that was eliminated or diminished during the chronic phase after infarction was significantly recovered after HGF-MSC, but not MSC, injection.

**Table 2:** Results of Dynamic ECG Recording for Each Group.

Parameter	HGF-MSC group	PBS group	MSC group
Time domain			
SDNN (ms)	_97.00±32.01_	_106.90±70.91_	_108.33±47.74_
CV (%)	_10.56±3.71_	_20.43±15.81_	_13.52±6.85_
SDANN (ms)	_84.22±29.41_	_81.60±48.85_	_100.89±51.27_
rMSSD (ms)	_35.22±9.23_^ab^	_12.60±2.88_	_21.44±10.97_ a
pNN50 (%)	_11.06±6.19_^ab^	_0.69±0.54_	_5.02±5.34_ a
Frequency domain			
LF	_44.29±16.12_^ab^	_93.59±23.33_	_57.76±8.02_ a
HF	_35.34±16.83_	_21.37±14.25_	_22.54±13.49_
LF/HF	_1.68±1.11_^ab^	_5.50±2.59_	_3.11±1.17_ a

a , *P*<0.05 vs. the PBS group;

b , *P*<0.05 vs. the MSC group.

### Programmed Electrical Stimulation (PES) of the Ventricles In Vivo

**Figure 6:**
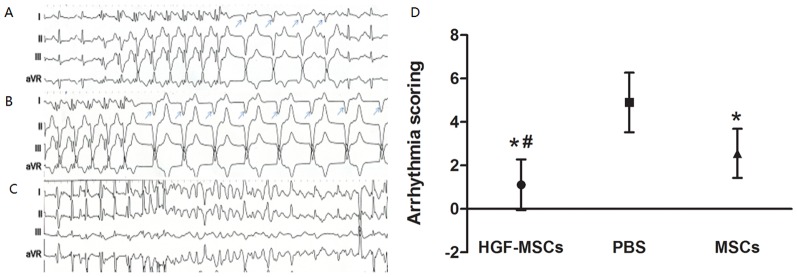
Electrophysiological examination to evaluate the propensity for VA. (A) Induced non-sustained ventricular arrhythmia. (B) Induced sustained ventricular arrhythmia. (C) Ventricular fibrillation evoked by additional stimuli. (D) The susceptibility to developing VA, as indicated by the obtained arrhythmia scores, was lower in the HGF-MSC-treated hearts than in the PBS- and MSC-treated hearts. *, *P*<0.05 vs. the PBS group; #, *P*<0.05 vs. the MSC group.

To further elucidate the physiological effects of HGF-MSC transplantation, PES was performed. Some pigs experienced non-sustained VT (Fig. 6A), sustained VT (Fig. 6B) or VF (Fig. 6C) during PES. While the arrhythmia score for evoked VA in the PBS group was quite high, this score was significantly reduced in the HGF-MSC and MSC groups (HGF-MSC group 1.11±1.17 vs. PBS group 4.90±1.37, *P*<0.01; MSC group 2.56±1.13 vs. PBS group 4.90±1.37, *P*<0.01) (Fig. 6D). This reduction in VA appeared to be significantly weaker in the MSC group than in the HGF-MSC group (MSC group 2.56±1.13 vs. HGF-MSC group 1.11±1.17, *P*<0.05) (Fig. 6D).

## Discussion

Previous studies have demonstrated that the survival of transplanted stem cells in vivo is dependent on the focal ischemic and hypoxic microenvironment [18]–[20]. Possible mechanisms leading to the death of engrafted cells include ischemic injury or activation of the apoptotic pathway [18]. Wu and colleagues found that HGF-MSCs induce substantial neovascularization in ischemic cardiac tissue [11], facilitating the long-term survival of these transplanted cells in the scarred region [21]. Our results provide further evidence of the survival of engrafted HGF-MSCs after transplantation and of the adaptation of these cells to the host environment. Moreover, our experiment indicated that the injected HGF-MSCs were able to differentiate into vascular endothelial cells or integrate into the developing vasculature, although there was insufficient evidence of cardiomyocyte differentiation [3], [21]–[23].

A series of investigations have revealed that certain cell types initiate apoptosis when they lose contact with the extracellular matrix [24]. The Bcl-2/Bax/caspase pathway has been shown to be involved in this pathological change. In our experiment, we detected increased Bcl-2 expression, decreased Bax expression, and a lower propensity for VA in HGF-MSC-treated hearts, compared with both MSC- and PBS-treated hearts. TUNEL staining has also demonstrated less apoptosis in the HGF-MSCs group than the other two groups. This apoptosis resistance effect could be due to either the enhancement of Bcl-2 expression or the inhibition of caspase activity [25], potentially via HGF-MSC transplantation. In addition, the HGF-MSC-treated IBZ regions displayed the greatest vessel density among the different groups, indicating that the focal blood flow and oxygen supply were substantially restored after HGF-MSC transplantation. Thus, the injected HGF-MSCs may serve as an anti-arrhythmic agent by attenuating MI-induced apoptosis and improving ischemic substrates.

Reentry, which is the cause of most VAs following MI in a chronic setting, is associated with electrical remodeling in the infarcted heart [26]. PES, the optimal assay for inducing reentry-mediated VA, has been widely applied to evaluate the risk of VA after cell therapy [2], [27]–[29]. It has been well established that the remodeling of gap junctions is an important pathological process in the occurrence of VAs. Anatomic or functional deficiency of Cx43, the major constituent of ventricular gap junctions, inevitably causes a delay in ventricular conduction, spontaneously inducing VA [30]. In this study, the MI-induced decrease in the area and lateralization of Cx43 expression in the IBZ was largely alleviated after HGF-MSC, but not MSC transplantation. These beneficial effects on Cx43 expression may lead to the amelioration of focal conduction and repolarization in the IBZ. Thus, it is not surprising that the HGF-MSC-treated hearts, which displayed the least electrical remodeling, exhibited the lowest propensity for VA among the three experimental groups.

Proliferative regeneration of Schwann cells and axons has been reported to be a consequence of necrotic injuries to the nervous system [31]. The coupling of increased sympathetic nerve sprouting with electrical remodeling of the myocardium may cause VT, VF and SCD [32]. Previous studies have found that MI induces an excessive and heterogeneous sympathetic nerve distribution in the IBZ [33]. According to our findings, compared with the PBS group, this heterogeneity was partially restored based on the significantly decreased sympathetic nerve sprouting and re-innervation observed in the HGF-MSC and MSC groups, especially in the former group.

In addition to the changes in the local nerve structure described above, we assessed the functional alterations at a global level between the different groups via dynamic ECG. The recordings obtained using this approach reflect the actual heart rate activity under social stress [34]. Finally, we detected a substantial enhancement of rMSSD and pNN50, along with a dramatic decrease in LF and LF/HF, in the HGF-MSC and MSC groups compared with the PBS group. This result is consistent with the findings of Ajijola, who demonstrated the occurrence of remodeling of the sympathetic innervations of the entire heart [35]. However, intramyocardial transplantation of HGF-MSCs significantly narrowed and minimized the MI-induced amplification of the sympathetic tone and reduction of the parasympathetic tone, indicating that HRV was clearly recovered in the HGF-MSC-treated hearts. Thus, HGF-MSC transplantation effectively suppressed the sympathetic tone and enhanced the parasympathetic tone, thereby recovering the homeostasis of the ANS. Numerous studies have shown that any intervention that elicits an increase in cardiac sympathetic activity also enhances the development of lethal cardiac arrhythmia [32], [36]–[39]. Moreover, interventions that reduce sympathetic activity are considered to protect against arrhythmia [39], [40]. Indeed, enhanced cardiac sympathetic activity has been associated with malignant VA in patients suffering from ischemic heart disease [41]. A study by Ajijola revealed that in a state of an enhanced sympathetic tone, a previously dormant channel in scar tissue may exhibit conduction during sympathetic activation, markedly altering the electrical properties of the scar and facilitating reentry-mediated VA [35], [42], [43]. Following HGF-MSC treatment, the risk for VA was remarkably reduced. Thus, attenuation of the heterogeneity of sympathetic nerve remodeling and rebalancing of the autonomic tone, thereby ameliorating neural remodeling, may represent the mechanisms underlying the HGF-MSC-mediated prevention of VA in a post-MI swine model.

In summary, the present study explored the antiarrhythmic effects of HGF-MSC transplantation after MI for the first time. Our findings suggested that intramyocardial transplantation of HGF-MSCs effectively reduces the susceptibility to developing VA, possibly through a mechanism involving the improvement of pro-arrhythmic substrates by ameliorating the electrical coupling between cardiomyocytes, attenuating neural remodeling, enhancing the blood and oxygen supply, and suppressing apoptosis in cardiac tissue. In short, HGF-MSC transplantation, which displays a robust restorative effect on cardiac function as well as potent anti-arrhythmic characteristics, is an ideal and promising strategy meriting further clinical investigation.

## Supporting Information

Figure S1 **TUNEL analysis in the infarcted border zone.** (A) HGF-MSC group. (B) PBS group. (C) MSC group. (D) Apoptotic index of different groups. TUNEL-positive nuclei were significantly decreased in the HGF-MSC group compared with other groups. * *P*<0.01 vs. the PBS group; # *P*<0.05 vs. the MSC group.
(TIF)

Text S1 **Supporting Materials and Methods.**
(DOCX)
