# Supplementary figures and images for: Hepatocyte Growth Factor Modification Enhances the Anti-Arrhythmic Properties of Human Bone Marrow-Derived Mesenchymal Stem Cells
Source: PLoS One. 2014 Oct 31;9(10):e111246. doi: 10.1371/journal.pone.0111246 (PMC4216066; doi:10.1371/journal.pone.0111246)

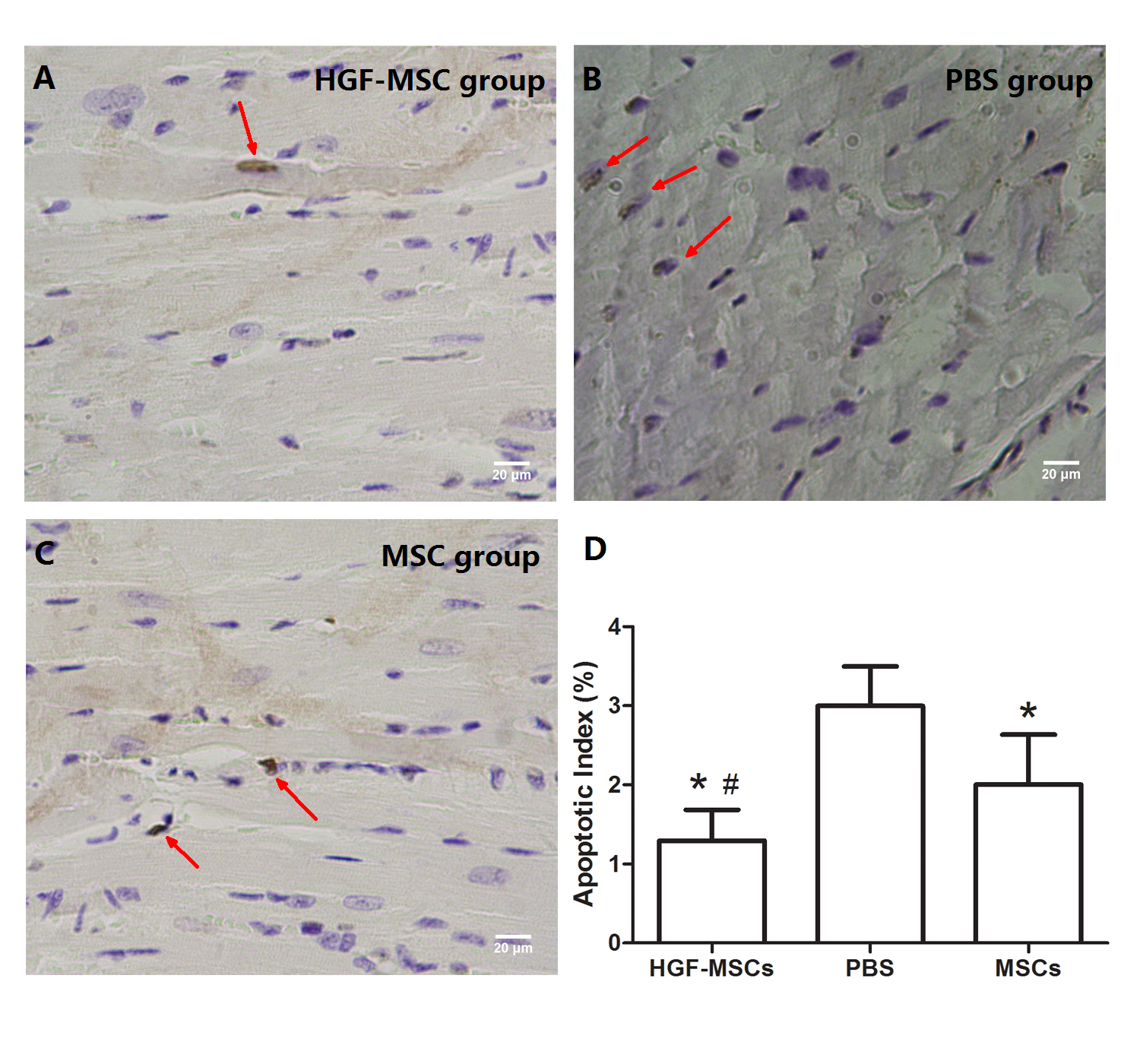

Supplement: Figure S1 — TUNEL analysis in the infarcted border zone. (A) HGF-MSC group. (B) PBS group. (C) MSC group. (D) Apoptotic index of different groups. TUNEL-positive nuclei were significantly decreased in the HGF-MSC group compared with other groups. * P<0.01 vs. the PBS group; # P<0.05 vs. the MSC group. (TIF) [file pone.0111246.s001.tif]
