# Supplementary material for: Hepatocyte Growth Factor Modification Enhances the Anti-Arrhythmic Properties of Human Bone Marrow-Derived Mesenchymal Stem Cells
Source: PLoS One. 2014 Oct 31;9(10):e111246. doi: 10.1371/journal.pone.0111246 (PMC4216066; doi:10.1371/journal.pone.0111246)
Supplement: Text S1 — Supporting Materials and Methods. (DOCX) [file pone.0111246.s002.docx]

**SUPPORTING INFORMATION**

**TITLE**: Hepatocyte growth factor modification enhances the anti-arrhythmic properties of human bone marrow-derived mesenchymal stem cells

**AUTHORS**:

Jian Zhang^1※^, Lin-Lin Wang^1※^, Wei Du^1^, Yi-Chao Yu^2^, Wei-Zhu Ju^1^, Yi-Long Man^1^, Xiao-Rong Li^1^, Yan Chen^1^, Zi-Dun Wang^1^, Wei-Juan Gu^1^, Feng-Xiang Zhang^1^, Hua Wang^3^, Chu-Tse Wu^3^, Ke-Jiang Cao^1^

**Supporting Materials and Methods**

**Two-Hour Dynamic Electrocardiography (ECG)**

Four weeks after cell engraftment, all of the pigs were sedated prior to the fixation of a digital dynamic electrocardiography monitor to the surface of their abdomen using adhesive tape. Dynamic ECG recordings were initiated when the swine were awake and conscious, and were performed for two hours. Then, the data were analyzed by a blinded operator using ECG Auto 1.5.7 software (EMKA Technologies). The signal waveforms, excluding misdetections and ectopic beats, were selected to calculate heart rate variability (HRV) as described by other investigators[[1](#_ENREF_1)]. The reference point for the temporal location of the R wave was recognized as the peak of the R spike. In the time domain, measurements were performed to obtain the following indices: the standard deviation of the normal-to-normal R-R intervals (SDNN), the standard deviation of the averaged normal-to-normal R-R intervals (SDANN), the root mean square of successive differences (rMSSD), the percentage of successive normal sinus R-R intervals longer than 50 ms (pNN50) and the coefficient of variance (CV; 100×SDNN/mean R-R interval). Furthermore, the power spectrum of the tachogram was assessed for the low-frequency spectra (LF; >0.5 Hz, <0.8 Hz), high-frequency spectra (HF; >0.8 Hz, <2.0 Hz) and the ratio of LF to HF (LF/HF) via frequency analysis.

**Electrophysiological Examination**

Once the dynamic electrocardiographic recordings were completed, the pigs were anesthetized again for monitor withdrawal and subsequent electrophysiological examination. A 6F electrode catheter for electrophysiological examination was advanced through the right femoral vein into the right ventricular apex, followed by programmed electrical stimulation (PES). To evaluate VA induction, the porcine heart was paced at a basic drive cycle length of 400 ms, which was combined with one to three additional stimuli (S2, S3 or S4) at shorter intervals. The progressive decrease in S1S2 was initially 10 ms and was 5 ms when approximating the refractory period. The protocols were performed identically for S2S3 and S3S4. The programmed ventricular stimuli were promptly terminated upon the initiation of a VA episode. A VA episode consisting of fewer than 15 heart beats was defined as non-sustained VA, whereas a VA episode consisting of 15 or more heart beats was considered to be sustained VA[[2](#_ENREF_2)]. Finally, the evoked potentials were classified and evaluated in accordance with a previously described arrhythmia scoring system[[3](#_ENREF_3)]: 0, non-inducible rhythm; 1, non-sustained tachyarrhythmia induced by three additional stimuli; 2, sustained tachyarrhythmia induced by three additional stimuli; 3, non-sustained tachyarrhythmia induced by two additional stimuli; 4, sustained tachyarrhythmia induced by two additional stimuli; 5, non-sustained tachyarrhythmia induced by one additional stimulus; 6, sustained tachyarrhythmia induced by one additional stimulus; 7, tachyarrhythmia induced during the basic drive cycle; and 8, the heart stopped before the basic drive cycle[[3](#_ENREF_3)].

**Western Blot and Immunofluorescence Analyses**

The housekeeping protein GAPDH (1:1000, sc-48166, Santa Cruz) was used as a standard internal reference. The BCA method was applied to determine protein concentrations. Each protein extract (40 μg) was subjected to electrophoresis on a 10% sodium dodecyl sulfate polyacrylamide gel (SDS-PAGE) and then transferred to a PVDF membrane. The membranes were incubated overnight at 4℃ with a primary antibody against Cx43 (1:1000, ab63851, Abcam), TH (1:200, sc-7847, Santa Cruz), GAP43 (1:200, sc-10786, Santa Cruz), AChE (1:200, sc-6431, Santa Cruz), Bcl-2 (1:200, sc-492, Santa Cruz) or Bax (1:100, sc-493, Santa Cruz). Subsequently, the membranes were incubated in a horseradish peroxidase-conjugated secondary antibody, followed by exposure of the protein bands using an ECL kit and the Image Lab system. The expression level of each protein was normalized to that of GAPDH using Image Lab software.

Alternatively, the samples collected from the IBZ were sliced into 4 μm sections and then routinely processed. Three sections of each heart were used for immunofluorescence analysis. The primary antibodies utilized for immunofluorescence staining were goat anti-TH (1:100, sc-7847, Santa Cruz), rabbit anti-GAP43 (1:100, sc-10786, Santa Cruz), goat anti-AChE (1:100, sc-6431, Santa Cruz), rabbit anti-Cx43 (1:500, ab63851, Abcam), mouse anti-von Willebrand factor (vWF)(1:400, ab68545, Abcam)and mouse anti-α-sarcomeric actin (α-SARC)(1:500, ab49672, Abcam). Nerve densities (at 200× magnification) were calculated using a computer-assisted image analysis system (Image-Pro Plus, Media Cybernetics, USA). The number of nerves and the area occupied by the nerves in each field were automatically computed. The density of the stained nerves was expressed as the nerve area divided by the total area examined (μm^2^/mm^2^). The nerve density of each section was calculated as the average of the 3 fields containing the highest nerve density[[4](#_ENREF_4)]. All of the recorded images of Cx43 staining were captured at 400X magnification, from which three visual fields per slice were randomly selected for further analysis using Image-Pro Plus. HGF-MSCs labeled with EdU and subsequently treated with an EdU kit were imaged under a fluorescence microscope as green fluorescence.

**Triphenyltetrazolium Chloride (TTC) Staining**

Upon sacrifice, the whole heart was quickly excised and placed in cooled saline (4°C) for 30 minutes. The myocardium of the LV was then cut into 5-10 mm slices in the short axis. All slices were immersed in a 500 ml solution of 1% TTC (Sigma-Aldrich, St. Louis, USA) made fresh in PBS (pH 7.4). Subsequently, slices were bathed at 37°C for 30 minutes and then ﬁxed in 4% formalin solution. The slices were grossly observed and then photographed under room light with a digital Canon camera.

1. Kruger C, Kalenka A, Haunstetter A, Schweizer M, Maier C, et al. (1997) Baroreflex sensitivity and heart rate variability in conscious rats with myocardial infarction. The American journal of physiology 273: H2240-2247.

2. Wang D, Zhang F, Shen W, Chen M, Yang B, et al. (2011) Mesenchymal stem cell injection ameliorates the inducibility of ventricular arrhythmias after myocardial infarction in rats. International journal of cardiology 152: 314-320.

3. Kang CS, Chen CC, Lin CC, Chang NC and Lee TM (2009) Effect of ATP-sensitive potassium channel agonists on sympathetic hyperinnervation in postinfarcted rat hearts. American journal of physiology Heart and circulatory physiology 296: H1949-1959.

4. Yuan MJ, Huang CX, Tang YH, Wang X, Huang H, et al. (2009) A novel peptide ghrelin inhibits neural remodeling after myocardial infarction in rats. European journal of pharmacology 618: 52-57.

**Supporting Data**

**The vWF-positive Vessel Counts in Different Groups**

|  | HGF-MSCs | PBS | MSCs |
| --- | --- | --- | --- |
|  | 20. | 7. | 11. |
|  | 30. | 11. | 18. |
|  | 28. | 6. | 18. |
|  | 26. | 5. | 16. |
|  | 26. | 10. | 15. |
|  | 26. | 9. | 14. |
|  | 24. | 4. | 14. |
|  | 24. | 9. | 14. |
|  | 23. | 9. | 13. |
|  |  | 10. |  |
| Average | $25.22\pm2.91$*^#^ | $8.00\pm2.36$ | $14.78\pm2.28$* |

* *P*<0.01 vs. the PBS group; ^#^ *P*<0.01 vs. the MSC group.

TUNEL Results

| Apoptotic Index(%) | HGF-MSCs | PBS | MSCs |
| --- | --- | --- | --- |
|  | 1.66 | 3.73 | 1.96 |
|  | 1.49 | 3.52 | 2.86 |
|  | 1.76 | 2.56 | 1.48 |
|  | 1.31 | 2.43 | 2.79 |
|  | 1.57 | 3.61 | 1.34 |
|  | 0.80 | 3.02 | 2.12 |
|  | 1.45 | 3.18 | 1.28 |
|  | 0.85 | 2.68 | 2.63 |
|  | 0.76 | 2.89 | 1.58 |
|  |  | 2.38 |  |
| Average | $1.29\pm0.39$*^#^ | $3.00\pm0.50$ | $2.00\pm0.63$* |

* *P*<0.01 vs. the PBS group; ^#^ *P*<0.05 vs. the MSC group.

**Data of Density of TH-positive Nerve**

| Nerve density  (μm^2^/mm^2^) | HGF-MSCs | PBS | MSCs |
| --- | --- | --- | --- |
|  | 3034 | 6403 | 3218 |
|  | 2865 | 7215 | 3203 |
|  | 2764 | 6554 | 3199 |
|  | 2733 | 6370 | 3303 |
|  | 3088 | 5838 | 3434 |
|  | 2888 | 6430 | 3257 |
|  | 2785 | 6773 | 3376 |
|  | 2798 | 6277 | 3664 |
|  | 2813 | 6865 | 3266 |
|  |  | 6600 |  |
| Average | $2863.11\pm122.48$^ab^ | $6532.50\pm371.26$ | $3324.44\pm150.08$^b^ |

^a^ *P*<0.01 vs. the PBS group; ^b^ *P*<0.01 vs. the MSC group.

**Data of Density of GAP43-positive Nerve**

| Nerve density  (μm^2^/mm^2^) | HGF-MSCs | PBS | MSCs |
| --- | --- | --- | --- |
|  | 1273 | 6174 | 2143 |
|  | 1259 | 6911 | 1651 |
|  | 1180 | 6492 | 1630 |
|  | 1108 | 5440 | 1495 |
|  | 1105 | 5571 | 1438 |
|  | 1060 | 7299 | 1365 |
|  | 1047 | 6969 | 1356 |
|  | 957 | 5812 | 2500 |
|  | 864 | 6994 | 2415 |
|  |  | 6532 |  |
| Average | $1094.78\pm133.23$^ab^ | $6419.40\pm647.53$ | $1777\pm453.13$^a^ |

^a^ *P*<0.01 vs. the PBS group; ^b^ *P*<0.01 vs. the MSC group.

**Data of Density of AChE-positive Nerve**

| Nerve density  (μm^2^/mm^2^) | HGF-MSCs | PBS | MSCs |
| --- | --- | --- | --- |
|  | 3345 | 2428 | 2299 |
|  | 2599 | 2112 | 2437 |
|  | 2379 | 2598 | 2440 |
|  | 2160 | 2189 | 2611 |
|  | 2149 | 2389 | 2336 |
|  | 2657 | 2011 | 2837 |
|  | 2438 | 2085 | 2504 |
|  | 2374 | 2392 | 2031 |
|  | 2380 | 1936 | 2159 |
|  |  | 2080 |  |
| Average | $2497.89\pm359.86$ | $2222.00\pm215.78$ | $2406.00\pm239.14$ |

Relative Protein Level

| TH | GAP43 | Cx43 | Group |
| --- | --- | --- | --- |
| 0.15 | 0.16 | 0.58 | 1 |
| 0.24 | 0.11 | 0.53 | 1 |
| 0.23 | 0.23 | 0.46 | 1 |
| 0.17 | 0.15 | 0.49 | 1 |
| 0.13 | 0.15 | 0.62 | 1 |
| 0.22 | 0.11 | 0.40 | 1 |
| 0.21 | 0.11 | 0.52 | 1 |
| 0.30 | 0.17 | 0.47 | 1 |
| 0.25 | 0.14 | 0.50 | 1 |
| 0.56 | 0.75 | 0.25 | 2 |
| 0.46 | 0.56 | 0.18 | 2 |
| 0.53 | 0.88 | 0.18 | 2 |
| 0.49 | 0.90 | 0.28 | 2 |
| 0.42 | 0.72 | 0.21 | 2 |
| 0.60 | 0.91 | 0.23 | 2 |
| 0.59 | 0.58 | 0.14 | 2 |
| 0.44 | 0.91 | 0.16 | 2 |
| 0.53 | 0.90 | 0.17 | 2 |
| 0.53 | 0.74 | 0.19 | 2 |
| 0.34 | 0.33 | 0.39 | 3 |
| 0.30 | 0.31 | 0.35 | 3 |
| 0.35 | 0.37 | 0.28 | 3 |
| 0.33 | 0.47 | 0.38 | 3 |
| 0.27 | 0.38 | 0.35 | 3 |
| 0.31 | 0.35 | 0.36 | 3 |
| 0.31 | 0.45 | 0.45 | 3 |
| 0.38 | 0.36 | 0.38 | 3 |
| 0.29 | 0.36 | 0.42 | 3 |

1 HGF-MSC group; 2 PBS group; 3 MSC group

Data of Dynamic Electrocardiogram

| Group | SDNN(ms) | CV(%) | SDANN(ms) | rMSSD(ms) | pNN50(%) | LF | HF | LF/HF |
| --- | --- | --- | --- | --- | --- | --- | --- | --- |
| 1 | 74 | 6.88 | 61 | 29 | 8.80 | 26.96 | 38.51 | 0.70 |
| 1 | 137 | 16.31 | 122 | 43 | 15.70 | 30.73 | 40.43 | 0.76 |
| 1 | 121 | 12.93 | 104 | 41 | 16.60 | 55.00 | 25.70 | 2.14 |
| 1 | 121 | 12.93 | 104 | 41 | 16.60 | 55.00 | 26.19 | 2.10 |
| 1 | 122 | 13.05 | 104 | 41 | 16.60 | 55.00 | 26.19 | 2.10 |
| 1 | 96 | 8.91 | 90 | 40 | 11.30 | 27.64 | 65.81 | 0.42 |
| 1 | 99 | 9.20 | 90 | 40 | 11.10 | 71.60 | 24.86 | 2.88 |
| 1 | 43 | 4.14 | 36 | 25 | 0.50 | 29.52 | 56.77 | 0.52 |
| 1 | 60 | 10.71 | 47 | 17 | 2.30 | 47.14 | 13.62 | 3.46 |
| 2 | 32 | 8.58 | 31 | 7 | 0.00 | 97.00 | 21.56 | 4.50 |
| 2 | 97 | 10.84 | 98 | 10 | 0.40 | 110.00 | 13.10 | 8.40 |
| 2 | 98 | 10.95 | 98 | 10 | 0.40 | 88.40 | 10.91 | 8.10 |
| 2 | 73 | 21.35 | 40 | 12 | 0.00 | 63.52 | 18.05 | 3.52 |
| 2 | 59 | 13.63 | 43 | 13 | 0.90 | 128.30 | 58.58 | 2.19 |
| 2 | 44 | 9.65 | 40 | 13 | 1.00 | 91.60 | 12.72 | 7.20 |
| 2 | 255 | 61.59 | 111 | 15 | 0.50 | 126.93 | 22.79 | 5.57 |
| 2 | 189 | 21.53 | 170 | 15 | 0.90 | 94.60 | 10.27 | 9.21 |
| 2 | 70 | 17.90 | 45 | 15 | 1.00 | 63.90 | 27.78 | 2.30 |
| 2 | 152 | 28.31 | 140 | 16 | 1.80 | 71.64 | 17.91 | 4.00 |
| 3 | 131 | 15.04 | 113 | 43 | 14.00 | 71.55 | 15.49 | 4.62 |
| 3 | 60 | 6.26 | 44 | 22 | 6.00 | 48.00 | 11.51 | 4.17 |
| 3 | 122 | 10.24 | 105 | 36 | 14.00 | 57.50 | 14.16 | 4.06 |
| 3 | 52 | 6.21 | 45 | 11 | 4.00 | 53.20 | 17.11 | 3.11 |
| 3 | 177 | 20.75 | 182 | 17 | 1.10 | 58.80 | 16.99 | 3.46 |
| 3 | 52 | 6.94 | 47 | 14 | 2.00 | 50.40 | 30.18 | 1.67 |
| 3 | 161 | 22.97 | 162 | 13 | 1.70 | 69.60 | 22.38 | 3.11 |
| 3 | 84 | 21.71 | 80 | 16 | 1.60 | 56.05 | 55.50 | 1.01 |
| 3 | 136 | 11.52 | 130 | 21 | 0.80 | 54.70 | 19.54 | 2.80 |

1 HGF-MSC group; 2 PBS group; 3 MSC group

Arrhythmia Scoring

| Score | HGF-MSCs | PBS | MSCs |
| --- | --- | --- | --- |
|  | 0 | 7 | 1 |
|  | 1 | 6 | 2 |
|  | 3 | 6 | 3 |
|  | 0 | 5 | 3 |
|  | 0 | 4 | 4 |
|  | 1 | 6 | 3 |
|  | 1 | 4 | 1 |
|  | 3 | 5 | 2 |
|  | 1 | 3 | 4 |
|  |  | 3 |  |
| Average | $1.11\pm1.17$*^#^ | $4.90\pm1.37$ | $2.56\pm1.13$* |

* *P*<0.01 vs. the PBS group; # *P*<0.05 vs. the MSC group.
